# Impact of type 2 diabetes on health expenditure: estimation based on individual administrative data

**DOI:** 10.1007/s10198-018-1024-9

**Published:** 2019-01-05

**Authors:** François-Olivier Baudot, Anne-Sophie Aguadé, Thomas Barnay, Christelle Gastaldi-Ménager, Anne Fagot-Campagna

**Affiliations:** 1 Université Paris-Est Créteil, ERUDITE, TEPP-FR CNRS 3435, IST-PE, Créteil, France; 2 Caisse Nationale de l’Assurance Maladie (Cnam), Paris, France

**Keywords:** Type 2 diabetes, Cost of illness, SNDS, Difference in differences, Exact matching, France, C23, H51, I18

## Abstract

Only limited data are available in France on the incidence and health expenditure of type 2 diabetes. The objective of this study, based on national health insurance administrative database, is to describe the expenditure reimbursed to patients newly treated for type 2 diabetes and the proportion of expenditure attributable to diabetes. The study is conducted over a 6-year period from 2008, the year of incidence of treated diabetes, to 2014. Type 2 diabetic patients aged 45 years and older are identified on the basis of their drug consumption. To estimate expenditure attributable to diabetes, a matched control group is selected among more than 13 million beneficiaries over 44 years old not taking antidiabetic treatment. The expenditure attributable to diabetes is estimated by two methods: simple comparison of reimbursed health expenditure between both groups, and a difference-in-differences method including control variables. The cohort of incident type 2 diabetic patients comprises 170,013 patients in 2008. Mean global reimbursed expenditure is €4700 per patient in 2008 and €5500 in 2015. Expenditure attributable to diabetes, estimated by direct comparison with controls, is €1500 in the first year. We, thus, observe a decrease in the following year due to decreased hospitalisations, and then expenditure increase by an average of 7% per year to reach €1900 in the eighth year after the initiation of treatment.

## Introduction

In the world, 422 million people suffered from diabetes in 2014 (versus 108 million in 1980), i.e., 8.5% of the adult population [11]. As shown by a systematic review [17], the economic burden of diabetes is considerable. In France, the diabetic population represented 4.4% of the population in 2009 [15]. In 2015, 5.4% of the main insurance scheme beneficiaries (private sector) take antidiabetic drugs. In 2006, 178,000 new cases were estimated [5]. As regard the growing prevalence of diabetes and the particular dynamics of diffusion of new and more expensive antidiabetic treatments [13], diabetes represents, in France, a high and growing burden for SHI (Statutory Health Insurance). It, therefore, appears essential to accurately estimate the cost attributable to diabetes, as well as the dynamics of this cost, i.e., the way in which the cost of diabetes varies as a function of the length of the disease.

Many medico-economic evaluations and cost projection studies focus on diabetes, using microsimulation methods (e.g., [2, 12]). However, we are not within that framework. Various approaches are used in cost-of-illness studies essentially to address two issues: the scope of the cost to be included and the comparator. Cost-of-illnesses studies can be classified into four categories [1, 4]: comprehensive global approach (all of the patients’ health expenditure); medicalized global approach (only directly related expenditure to the disease); incremental regression approach (cost estimation from a model); incremental approach with a control group (the cost is calculated by the difference). For example, the comprehensive global approach was used on German [18] and French data [4]. An Italian study estimated the direct and indirect costs of diabetes by combining Italian epidemiological data with cost data derived from the literature [9]. The incremental approach with a control group is used by Mata-Cases et al. [10]. De Lagasnerie et al. [4] also combined this approach with the medicalized global approach. All of these studies are based on the populations of prevalent diabetic patients and only one has been conducted on French data. Tao et al. [20] conducted a study on a cohort of patients with newly diagnosed type 1 diabetes. They used a control group composed by propensity score matching to estimate the expenditure attributable to type 1 diabetes. Only one study has, therefore, been conducted on French data, Therefore, the only recent study on a cohort of newly diagnosed diabetic patients concerned type 1 diabetes. Furthermore, the issue of cost estimation of a beginning disease and the subsequent growth of costs according to progression of the disease remain very poorly studied in the international literature.

Therefore, to deeply analyse the onset of diabetes, the *Caisse Nationale de l’Assurance Maladie* (SHI) decided to set up an ad hoc cohort of newly treated type 2 diabetic patients. The implementation of this cohort allows studying the impact of this disease and its complications on health expenditure and its evolution. The objective of this study is then to estimate the excess expenditure reimbursed by SHI between 2008 and 2015 for a population newly treated for diabetes in 2008, based on panel data and a DiD (difference-in-differences) method with exact matching.

This study presents a number of original features. First, data come from the French SHI database (*Système national des données de santé* or SNDS). In 2008, SNDS contains individual-level information from reimbursement claims for all the French population, allowing, for our study, the constitution of a large cohort of more than 170,000 individuals. Second, recent studies use a cross-sectional approach to study all prevalent diabetic patients, while our approach focuses on a cohort of patients newly treated for type 2 diabetes (cases). We can then estimate the course of reimbursed expenditure over time, allowing to observe the effect of the progressive onset of complications. Third, the control group is very large (more than 520,000 nondiabetic individuals) and presents similar characteristics to the treated group by performing exact matching method. This control group is used to analyse the proportion of reimbursed expenditure specifically due to the onset of type 2 diabetes by using the DiD method.

## Methodology

### Data

Data used come from the SNDS, which contains comprehensive healthcare reimbursement data for all individuals covered by the SHI. This database has been described by Tuppin et al. [19] and contains in particular: demographic characteristics concerning beneficiaries; diagnoses information for beneficiaries under the for “ALD” status which waives co-payments related to some specific long-term diseases, but also for hospitalisation stays, sick leaves (lasting 6 months and longer), and disability pensions; the dates of death of beneficiaries, when applicable; reimbursement data: primary care (types and dates of procedures performed by private physicians, dentists, etc.; medical devices and associated services, reimbursed drugs, etc.); private and public-sector hospitalisations; cash benefits (sick leave, disability pensions, workers’ compensation, occupational disease, or death benefits).

All data are anonymous and individually linkable. The use of a medical administrative claim database ensures major statistical power and provides highly representative samples. The SNDS database is, therefore, used to guide public decisions and can be used for international comparisons.

### Scope of the study

This study is based on the whole population of France [Metropolitan France and overseas departments and territories (DOM–TOM)] covered by the main health insurance scheme (including only private-sector salaried employees, excluding officials and students) who received at least one healthcare reimbursement (at least €1) each year (unless they had died) from 2008 to 2015. In 2008, of the 64 million inhabitants in France, 50 million people (i.e., 77% of the population) were covered by the general scheme. Health expenditure reimbursed by SHI during the 2006–2015 follow-up period; both groups (cases and controls) are studied.

### Scope of expenditure

This study is conducted from the SHI perspective (payer). We consider all expenditure reimbursed by SHI (reimbursements from other health insurance schemes, private health insurance, or the final out-of-pocket are not included) expressed in real terms (deflated by consumer price index).

Expenditures are aggregated according to the following expenditure items: primary care (mainly healthcare professional fees, reimbursements of drugs and medical devices, and laboratory tests), public- and private-sector medicine, surgery, obstetrics (MSO) hospital care, and cash benefits (essentially sickness benefits and disability pensions). Each type of expenditure is presented for each year from 2006 to 2015, except for public-sector hospitalisations for which costs of hospital stays are only available from 2008 onwards. Expenditures concerning hospital at home (HAH) and outpatient department (OPD) visits and procedures, aftercare and rehabilitation (Rehab) stays, and psychiatric hospital care were excluded throughout the study period, as cost data were not available for the entire study period.

### The cohort of incident-treated type 2 diabetic patients in 2008

Diabetic patients are identified from reimbursement data concerning diabetes-specific treatments. A patient is defined as being diabetic when he/she receives at least three dispensing of antidiabetic drugs [oral antidiabetics (OAD) or insulin] during the year, or at least two in the case of reimbursement of at least one large pack-size OAD. A diabetic person is considered to be incident in a calendar year when he/she received at least three (or two in the case of a large pack size) antidiabetic drugs in that year but not during the previous 2 years. Antidiabetics’ drugs are those belonging to class A10 of the Anatomical Therapeutic Chemical (ATC) classification with the exception of benfluorex. Only people 45 years and older in 2008 are included in the cohort, to select as specifically as possible only those patients with type 2 diabetes. For the same reason, women hospitalised for gestational diabetes in 2008 are also excluded. Finally, people presenting at least 1 year with no reimbursed health expenditure from 2009 to 2015 and who had not died are also excluded.

Mean reimbursed expenditure corresponds to the annual mean (calendar years) per consumer.

The cohort of incident type 2 diabetic patients in 2008 comprises 170,013 individuals with a mean age of 63.5 years (median 62 years). Men represented 52.6% of the cohort and, on average, were younger than women (62 years and 65 years, respectively). Patients of the cohort belonged to more deprived social categories than the general population, as 8.5% of the cohort received at least one universal complementary health insurance reimbursement in 2008, versus 5.7% in the general population [6]. In addition, 68% of the cohort is eligible, at the end of 2008, for exemptions of co-payment due to ALD status, with diabetes-specific ALD status in 43% of cases, confirming that attribution of ALD status is not a good marker to monitor the incidence of type 2 diabetes. At 31 December 2015, 15% of the cohort had died.

### The control group

Now, we aim to determine the proportion of this expenditure that can be attributed to type 2 diabetes. This study is designed to evaluate the impact of onset of type 2 diabetes on the level of expenditure compared to that of a not treated population. Estimation of the excess cost related to diabetes constitutes part of the general framework of the Rubin causal model [16].

Matching methods are used to correct for selection effects between treated and non-treated patients by controlling for observable differences to identify the causal effect of treatment. We assume that all determinants of selection of treated patients are captured by observable differences between both groups. The identifying assumption used to estimate this causal effect is that, depending on the observable characteristics, allocation or non-allocation of treatment is independent of its potential effects (Conditional Independence Assumption). Expenditure attributed to a patient of the treated group is counterfactual to the expenditure attributed to a patient of the non-treated group if he or she had not been treated and vice versa.

Exact matching is the simplest method, which consists of matching each individual of the control group with one or several “twins”. Subject to complying with the common support assumption, a twin presenting strictly identical characteristics can be found in the control group in the case of discrete matching variables [7].

The control group is built by exact matching. The objective is to take into account observable characteristics that could partly explain the excess cost without inevitably being directly related to type 2 diabetes. For example, major comorbidities or a lower level of education in the group of diabetic patients could explain an increased cost. Matching can, therefore, cancel the effect of the variables used on the variable of interest.

The control group is constituted in several steps. First of all, a potential control group is composed by the entire French nondiabetic population covered by the strict general scheme over 44 years old in 2008 and reimbursed for healthcare each year from 2008 to 2015 (or who had died). Diabetic patients excluded from this population were those who had received at least three dispensing of antidiabetic drugs (or two when a large pack size was dispensed) in the same year from 2006 to 2015, as well as women hospitalised for gestational diabetes from 2006 to 2015. Controls were then randomly selected from this population on the basis of all matching variables.

The potential control group is a similar group of treated patients with the exception of the criterion concerning diabetes. It comprises more than 13 million nondiabetic beneficiaries.

#### Matching method and results

For each case, three controls are selected at random, when available. Matching variables consist of sociodemographic variables (year of birth, sex, quintile of social deprivation index of the last place of residence in 2008, and a place of residence indicator in DOM–TOM in 2008), a proxy of risk factors for diabetes, and the decile of resource consumption in 2007 among patients of the incident diabetic cohort. Matching variables must have an impact on the variable of interest (reimbursed expenditure), but must not be influenced by treatment (diabetes). The values used for the proxy of risk factors variable and the decile of resource consumption are those of the year preceding the diagnosis of diabetes and can, therefore, not have been influenced by onset of the disease.

The social deprivation index is calculated for each town of metropolitan France for 2009 using the method described by Rey et al. [14]. The quintile of social deprivation index of the last place of residence in 2008 for each individual is used for matching. As this index is not available for DOM–TOM, a DOM–TOM indicator variable was added. The places of residence used corresponded to those in 2008, but the deprivation index calculated is that for 2009. A sixth modality is attributed to individuals with a missing social deprivation index in 2007 and 2008.

Some diabetes risk factors (such as overweight) can have an impact on future health expenditure. Failure to take these risk factors into account could result in a bias. For example, there would be a risk of matching future diabetic patients with a mean weight higher than that of the control, and a proportion of expenditure attributable to overweight would be attributed to diabetes. In the absence of sufficiently detailed medical data in the database, we use a proxy for certain diabetes risk factors. To build this variable, a value of 1 is attributed when the individual received at least three dispensing of antihypertensive or lipid-lowering drugs in 2007 (or at least two dispensing when at least one large pack size was dispensed). If the individual consumed healthcare reimbursed by the general scheme in 2007 but not the various drugs considered, a value of 0 is attributed to the variable. When the individual did not consume any healthcare reimbursed by the general scheme in 2007, a third modality is attributed.

Finally, SHI reimbursable expenditure in 2007 for incident diabetic patients in 2008 is also considered. SHI reimbursable expenditure corresponds to the sums effectively paid by primary health insurance funds, to which must be added the out-of-pocket expenses for patients and/or private health insurance in the limit of approved tariffs and regulatory nomenclatures. The expenditure includes all primary care, cash benefits, and private-sector hospitalisations (but public-sector hospitalisations’ reimbursable expenditure is not included). Both groups are classified into deciles of reimbursable expenditure calculated from this expenditure. An 11th modality is attributed to individuals with no general scheme reimbursable healthcare in 2007. These individuals may have consumed healthcare reimbursed by other schemes and may not inevitably be non-consumers.

Of the 170,013 individuals of the cohort, 170,005 are matched with three controls. The control group is, therefore, composed of 510,022 nondiabetic individuals. Exact matching ensures a perfect balance in terms of matching variables. As shown in Table 1, matching allows to partially control for the imbalance observed in terms of observable heterogeneity. In particular, the proportion of patients aged 45–60 years covered by CMU-C (universal medical coverage) in 2007 was twofold higher among the matched cohort than among the potential controls. This difference is only very slightly reduced (0.9 percentage point) after matching.

**Table 1:** Descriptive data before and after matching. Source: SNDS

	Matched cases	Potential controls	Matched controls
Matching variables			
Mean age in 2008	63.5 years	61.8 years***	63.5 years
Proportion of men	52.6%	40.8%***	52.6%
Place of residence in the most deprived quintile	21.3%	25.5%***	21.3%
Place of residence in the least deprived quintile	20.3%	16.0%***	20.3%
DOM–TOM residents	3.8%	1.8%***	3.8%
Control variables			
Percentage of patients hospitalised (all reasons)	27.1%	21.9%***	23.8%***
Percentage of patients covered by CMU-C (45–60 years)	13.8%	6.1%***	7.0%***
At least one cardiology ALD (disabling stroke, chronic peripheral artery disease, severe heart failure, hypertension, or coronary heart disease)	17.7%	11.1%***	14.2%***
At least one ALD (excluding multiple diseases)	38.2%	26.0%***	28.7%***
Number of individuals	170,010	13,413,578	510,022

Field: French population insured by general scheme of SHI in 2008. Interpretation: In 2008, the average age was 63.5 years for the matched cases, 61.8 years for the potential controls, and 63.5 years for the matched controls

***p value < 0.001 (differences between matched cases and controls)

#### Estimation of the excess cost attributable to diabetes

The objective of the Rubin causal model is to estimate the counterfactual cost of a diabetic individual (i.e., the cost of this individual without diabetes) [16]. In practice, the excess cost attributable to diabetes can be estimated by comparing a group of people with diabetes to a group with strictly similar characteristics but without diabetes. Simple comparison of treated and non-treated individuals (naive estimation) leads to a high risk of bias due to the non-random distribution of diabetes among the individuals [16]. Two distinct methods are thus used to estimate the excess cost attributable to diabetes: simple difference and DiD.

First, the excess cost initially corresponds to the difference in reimbursed expenditure between the both groups. This method is based on the assumption that matching made the two groups strictly identical, but only related to the matching variables. The mean expenditure for each type of expenditure is determined between the healthcare-consuming twins of the same case, to build a “mean” synthetic control. Consequently, the sample sizes of two groups are identical for simple differences analysis. Differences are calculated by group and not by pair.

Second, several DiD techniques were then used, in addition to matching. The DiD method assumes that the treatment indicator (being diabetic) is exogenous, i.e., not correlated with individual effects [7]. The first difference is determined for each individual’s expenditure before and after the onset of diabetes in the two groups, to eliminate the fixed individual heterogeneity over time. For example, this method neutralizes the effect of differential preference of healthcare consumption between individuals and certain genetic susceptibilities. The second difference is determined between the individual’s mean reimbursed expenditure in the two groups to neutralize unobserved temporal effects (temporal heterogeneity); for example, increased use of drugs not related to diabetes in the population.

This method also allows the use of a number of control variables to correct the observed heterogeneity. The control variables are those not used for matching. These variables could have an impact on reimbursed expenditure, without being affected by the onset of type 2 diabetes. Eight variables are selected: the presence of at least one CMU-C reimbursed expenditure in 2007; at least one hospitalisation in 2007, at least one healthcare reimbursement in 2007 in the context of:

- ALD 1 (disabling stroke), 3 (chronic peripheral artery disease), 5 (heart failure), or 12 (hypertension);
- ALD 11 (haemophilia and clotting disorders);
- ALD 15 (Alzheimer’s disease) or 16 (Parkinson’s disease);
- ALD 23 (psychiatric illness);
- ALD 30 (malignant tumour);
- another ALD.

The reference year chosen for DiD analysis is 2007, 1 year before the onset of type 2 diabetes. Estimations are performed for each year from 2008 to 2015, and for four variables of interest: reimbursed primary care expenditure, reimbursed private-sector hospitalisation expenditure, cash benefits, and total reimbursed expenditure (primary care, private-sector hospitalisations, and cash benefits). Moreover, public-sector hospitalisations are not taken into account (cost data are unavailable for 2007).

For simple differences, as well as DiD, individuals not consuming any healthcare or who died are treated as follows:

- before 2008, non-consumers did not receive any reimbursements from the strict general scheme: the value zero is attributed to each type of expenditure;
- from 2008 onwards, all non-consumers died are excluded from the analysis from the year following their death:
  - when a matched case died, the associated controls are excluded;
  - when a control died, the cases are only excluded when all associated controls died; otherwise, the mean expenditure of the surviving controls was used.

At 31 December 2015, 12.6% of controls died versus 14.7% of cases.

In 2006, 94% of controls are consumers versus 88% of cases. In 2007, 93% of individuals in both groups are consumers (due to a specific modality in matching variables). In 2008, all individuals are consumers (as determined by inclusion criteria). After 2008, due to the exclusion of non-consumers survivors, the number of consumers is equal to the number of surviving patients.

### Econometric strategy

Formally, the model used for calculation of the reimbursed expenditure attributable to diabetes (DiD) was as follows:

$${y_{it}}={e_t}+{e_i}+\delta {T_{it}}+{X_{it}}\beta +{u_{it}},$$

where *e*_*t*_ are the time fixed effects capturing the temporal changes affecting all groups, *e*_*i*_ are the individual fixed effects, *T*_*it*_ is the treatment indicator (equal to 1 for diabetic patients and 0 for nondiabetic patients), *X*_*it*_ represents the control variables, and *u*_*it*_ is the perturbation. *y*_*it*_ is the expenditure reimbursed to patient *i* at date *t*.

## Results

### Growth of reimbursed expenditure of incident diabetic patients in 2008

The mean total expenditure for a diabetic patient is about €4700 in 2008 and about €5500 in 2015. In 2008, 50% of expenditure is related to primary care, 38% to hospitalisations, and 12% to cash benefits. The proportion of expenditure related to hospitalisations in 2009 decreased to 33%. This decline is compensated by the increased proportion related to primary care (54% of reimbursed expenditure). Primary care and hospitalisations expenditure regularly increase from 2010 onwards, by an average of 3% and 4% per year, respectively. Cash benefits decreased throughout the study period. Mean reimbursed expenditure per year is shown in Table 2.

**Table 2:** Mean reimbursed expenditure per year. Source: SNDS

Year	2006	2007	2008	2009	2010	2011	2012	2013	2014	2015
Primary care										
General practitioner care	€137	€145	€183	€182	€178	€181	€179	€176	€177	€207
Other specialist care	€167	€170	€204	€203	€206	€211	€209	€212	€220	€227
Dental care	€54	€53	€52	€52	€50	€50	€48	€48	€48	€49
Midwifery	€0	€0	€0	€1	€0	€0	€0	€0	€0	€0
Physiotherapy	€72	€79	€90	€97	€99	€100	€106	€114	€120	€125
Nursing care	€98	€126	€270	€327	€346	€369	€407	€446	€489	€531
Other paramedical care	€5	€5	€7	€8	€9	€9	€10	€11	€13	€14
Clinical pathology	€84	€91	€135	€131	€133	€131	€127	€127	€127	€128
Drugs	€760	€799	€1021	€1146	€1182	€1197	€1183	€1164	€1252	€1207
Medical devices and services	€116	€142	€258	€276	€290	€291	€299	€317	€342	€365
Transportation	€87	€99	€134	€141	€149	€155	€165	€179	€193	€210
Other primary care	€8	€8	€8	€8	€9	€9	€9	€9	€9	€10
Total primary care	€1588	€1718	€2362	€2571	€2651	€2703	€2741	€2803	€2989	€3074
Hospital care										
Total private sector	€399	€396	€476	€476	€476	€486	€500	€514	€545	€570
Total public-sector	NA	NA	€1327	€1109	€1107	€1127	€1199	€1215	€1302	€1408
Total (public and private)	NA	NA	€1803	€1585	€1583	€1613	€1698	€1730	€1847	€1977
Cash benefits										
Sickness benefits and work accidents/occupational diseases	€281	€282	€321	€276	€232	€199	€177	€159	€152	€142
Disability benefits	€283	€258	€256	€301	€314	€319	€331	€326	€322	€314
Total cash benefits	€564	€540	€577	€576	€546	€518	€508	€486	€475	€456
Total expenditure										
Total expenditure other than hospitalisations	€2632	€2744	€3527	€3734	€3793	€3838	€3889	€3948	€4158	€4260
Total expenditure	NA	NA	€4742	€4733	€4779	€4835	€4947	€5018	€5311	€5507

NA: public-sector hospitalisation costs are not available for 2006 and 2007. Field: expenses reimbursed by the general scheme of SHI. Interpretation: in 2006, the SHI reimbursed an average of €137 per patient for the care of general practitioners (having used at last one reimbursed care reimbursed in 2006)

**Fig. 1:**
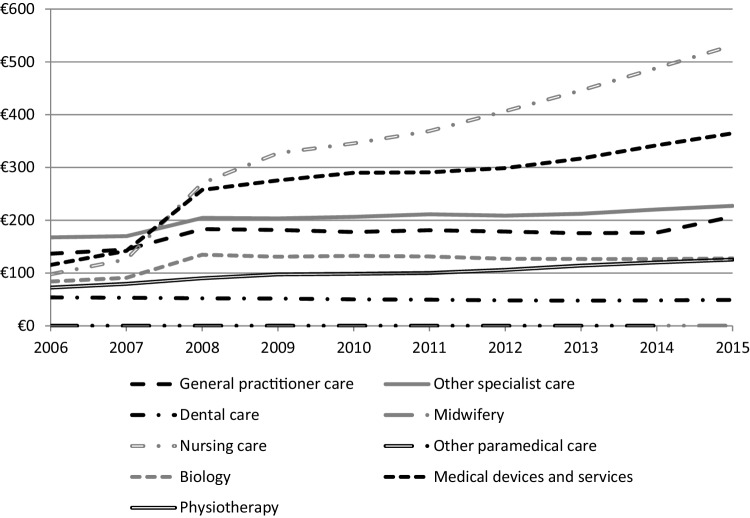
Growth of expenditure per patient and by type of primary care expenditure, excluding drugs, from 2006 to 2015 (diabetic cohort). Field: expenses reimbursed by the general scheme of SHI, excluding drugs. Source: SNDS

A very significant increase in reimbursed primary care expenditure is observed in 2008, the year of incidence of treated type 2 diabetes: reimbursed expenditure increased from €1700 per patient in 2007 to €2400 per patient in 2008, i.e., a 38% increase. The most dynamic drivers of primary care expenditure are drug reimbursements (which increased by 28% in 2008 compared to 2007 and accounted for 34% of growth for that year), nursing care (which increased by 115% in 2008 and accounted for 22% of growth), and medical devices and services (which increased by 81% in 2008 and accounted for 18% of growth). Cost trends are shown in Fig. 1 (excluding drug reimbursements). The largest proportion of primary care expenditure is due to drugs (43% in 2008; 39% in 2015).

### Estimation of the excess cost attributable to treated type 2 diabetes by the simple differences method

The mean annual growth rate (MAGR) of expenditure from 2008 to 2015 is 1% in controls and 2% in cases. The excess cost (i.e., difference of mean reimbursed expenditure between cases and controls) is, therefore, €1500 a year of incidence of treated type 2 diabetes. This excess cost then decreases to €1300 in the second year (− 13%), due to the decreased excess cost related to hospitalisations, which corresponded to the lowest excess cost measured throughout the study period. From 2010, the MAGR was 7% and the excess cost related to diabetes was €1889 in 2015. In 2008, 51% of the excess cost is related to primary care, 41% to hospital care, and 8% to cash benefits. In 2009 and 2010, the excess cost related to hospitalisations decreases in favour of primary care (68% and 73% of excess cost, respectively), before increasing again in 2011 to reach 28% in 2015 (67% for primary care and 5% for cash benefits). Table 3 lists the differences of expenditure between cases and controls by year.

**Table 3:** Differences of reimbursed expenditure between cases and controls (simple difference). Source: SNDS

Year	2006	2007	2008	2009	2010	2011	2012	2013	2014	2015
Primary care										
Total primary care	€175	€96	€756	€886	€958	€1005	€1050	€1104	€1222	€1260
Hospital care										
Total private sector	€41	− €37	€54	€40	€44	€55	€70	€83	€102	€120
Total public-sector MSO	NA	NA	€552	€258	€208	€228	€315	€305	€366	€409
Total MSO (public and private)	NA	NA	€607	€298	€251	€282	€384	€387	€468	€530
Cash benefits										
Sickness benefits and work accidents/occupational diseases	€31	€19	€97	€66	€32	€14	€9	€9	€9	€9
Disability benefits	€41	€16	€22	€46	€66	€77	€84	€87	€89	€89
Total cash benefits	€72	€35	€119	€112	€97	€91	€93	€96	€98	€98
Total expenditure										
Total expenditure (excluding public-sector hospitalisations)	€310	€106	€956	€1051	€1119	€1175	€1247	€1316	€1451	€1519
Total expenditure (hospitalisations: MSO only)	NA	NA	€1482	€1295	€1307	€1378	€1528	€1588	€1788	€1889

Scope: expenses reimbursed by the general scheme of SHI. Interpretation: in 2008, primary care expenditure reimbursed to a diabetic patient was €756 higher than that reimbursed to a patient of the control group

#### Primary care

**Fig. 2:**
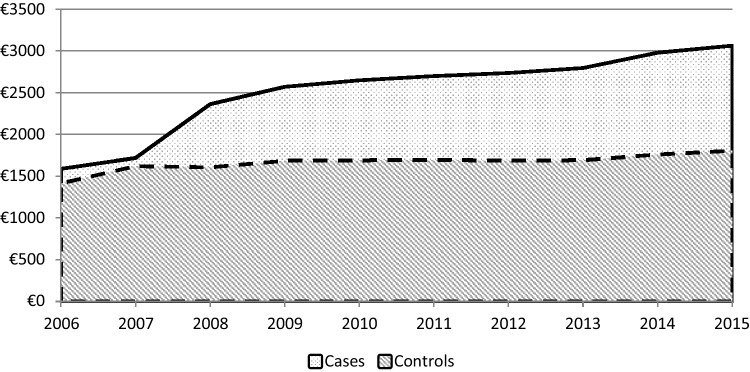
Comparison of mean reimbursed primary care expenditure per year for cases and controls. Scope: expenses reimbursed by the general scheme of SHI. Source: SNDS

Figure 2 presents the growth of mean primary care expenditure. The similarity of expenditure between cases and controls in 2006 and 2007, before onset of diabetes, shows the quality of matching. From 2008, the excess cost related to diabetes is €756 (39% due to drugs, 21% due to nursing care, 18% due to reimbursed medical devices and services, and 7% due to general practitioner visits).

From 2009, the MAGR is 11% and the excess cost reaches €1260 in 2015. During the period 2008–2015, the increases in primary care are drugs (32%), nursing care (20%), and medical devices and services (12%).

Comparative analysis of the growth of expenditure can be used to estimate the excess cost related to diabetes, especially for certain types of expenditure that would increase even in the absence of diabetes. In particular, “nursing care” expenditure increases in the general population during the follow-up. Although a strong growth of nursing care expenditure noticed from 2008 to 2015, the impact of the incidence of diabetes cannot be demonstrated. Indeed, this trend appears at 2007 (29% increase compared to 2006). In the controls, nursing care expenditure increases by only 17% in 2008 compared to 2009 (while it increases by 117% in 2008 in the cases), with an MAGR of 9% starting from 2009 (10% in cases). The excess cost of nursing care expenditure in cases compared to controls is, therefore, €160 in 2008 with a MAGR of 11% starting from 2009 to reach €329 in 2015. Similarly, medical devices and services expenditure increase for both cases and controls, but with a growth of 81% for cases in 2008 (followed by a mean annual growth of 5%) versus 15% for controls (followed by a mean annual growth of 5%). This growth in medical devices and services expenditure results in an excess cost of €133 in 2008 for cases compared to controls, with a subsequent mean annual growth of 5% to reach €187 in 2015. Finally, drug expenditure presents different patterns in both groups. From 2009 onwards (compared to the previous year up until 2015), mean expenditure increases by an average of 3% per year for cases, while it decreases by an average of 1% per year for controls. The excess cost related to drugs was €297 in 2008, and then increases by an average of 9% per year to reach €523 in 2015.

#### Hospitalisations

The mean excess cost related to private- and public-sector MSO (medicine, surgery, and obstetrics) hospitalisations for cases compared to controls is €607 in the first year. The excess cost decreases by more than 50% to €298 in the second year. Differential analysis, therefore, shows that the observed decrease in mean hospitalisation expenditure does not have a conjunctural causation, but corresponds to a true decrease in hospital expenditure during the year following the year of incidence. No significant difference in trends is observed between public-sector and private-sector hospitalisations. On average, the public sector represents more than 80% of all MSO hospitalisation expenditure from 2008 to 2015. Finally, the mean annual growth rate of the excess cost related to MSO hospitalisations is 2% from 2009 to 2015.

#### Cash benefits

The excess cost related to cash benefits is €119 in 2008, and then remains stable from 2009 to 2015 (mean of €98). The structural reduction of cash benefit reimbursements is due to ageing, but has no impact on the excess cost because. Indeed, as a result of matching, controls are exactly the same age as cases and calculation by differences neutralized the effect of ageing.

### Estimation of excess cost by DiD

Expenditure used for estimation of the excess cost by the DiD method comprise: primary care, private-sector MSO hospitalisations exclusively, and cash benefits. Public-sector hospitalisations are excluded due to data unavailability in 2007. Values of the coefficients of interest of DiD are presented in Table 4.

**Table 4:** Values of the coefficients of interest of DiD (*δ*), by year, for total reimbursed expenditure (excluding public-sector hospitalisations). Source: SNDS

	2008	2009	2010	2011	2012	2013	2014	2015
Total expenditure	€896	€978	€1044	€1102	€1174	€1243	€1385	€1452
Primary care	€695	€816	€889	€940	€987	€1044	€1169	€1205
Private-sector hospitalisations	€91	€56	€63	€75	€98	€107	€123	€153
Cash benefits	€99	€94	€81	€75	€79	€81	€82	€82

Scope: reimbursed primary care, private-sector MSO hospitalisations, and cash benefits. Interpretation: in 2008, the presence of incident-treated diabetes in 2008 increased the total reimbursed expenditure (excluding public-sector hospitalisations) by an average of €896 compared to nondiabetics

The excess cost attributable to the incidence of treated type 2 diabetes, all other things being equal, is €896 for the first year of treatment. The growth of this excess cost remains stable over time with a mean annual growth of 7% from 2009 to 2015.

The excess cost calculated by DiD for primary care is €695 in 2008, close to simple difference’s finding (€756). The mean annual growth is 8%. The excess cost of private-sector hospitalisation expenditure is €91 in the year of incidence of treated type 2 diabetes with a mean annual growth of 10%. The excess cost related to cash benefits remained fairly stable from 2008 to 2015 (mean decrease of − 2%) between €75 and €100 per year.

### Simple difference and DiD methods: a comparison

The results of the two methods are compared (Table 5). Only minor differences are observed for primary care and total expenditure (excluding public-sector hospitalisations), but much more marked differences are observed for private-sector hospitalisations and cash benefits. The smaller differences for total expenditure are probably, at least partially, due to the use of the decile of total expenditure in 2007 (excluding public-sector hospitalisations) which is used as a matching variable. Differences can also be explained by the effect of control variables (an ALD patient for cardiovascular risk would probably be at greater risk of being hospitalised for myocardial infarction), and by correction of the time-constant individual heterogeneity (for example, for cash benefits, much of which corresponded to pensions allocated over long periods for reasons not necessarily related to diabetes).

**Table 5:** Comparison of the results obtained by simple difference and DiD methods (mean excess cost from 2008 to 2015)

Method	Simple difference	DiD	Percentage difference
Primary care	€1030	€968	− 6
Private-sector hospitalisations	€71	€96	35
Cash benefits	€101	€84	− 16
Total (excluding public-sector hospitalisations)	€1202	€1159	− 4

## Discussion, limitations, and prospects

### Discussion of the results

The growth of expenditure refers the growing prevalence of diabetes. The onset of treated diabetes in 2008 naturally resulted in a clear increase of drug expenditure in 2008. The first-line treatment recommended by the French National Authority for Health at the beginning of the disease is metformin monotherapy. Oral treatment is intensified (dual therapy, followed by triple therapy) during the subsequent course of the disease. Insulin therapy may also be required when patients fail to respond to oral antidiabetics and when blood glucose targets are not achieved.

The growing number of insulin-treated patients has an impact on drug expenditure, but also expenditure related to nursing care and medical devices and services, essentially comprising self-treatment medical devices and blood glucose self-monitoring. The marked increase in laboratory test expenditure can be explained by the tests recommended for the diagnosis and follow-up of patients with type 2 diabetes: glycosylated haemoglobin (HbA1c) and blood glucose, as well as lipid assessments and renal function tests.

MSO hospitalisation expenditure presents different patterns between the public and private sectors. A moderate rise in private-sector expenditure was observed in 2008. Due to the absence of data for the public sector in 2006 and 2007, the growth of expenditure in 2008 cannot be determined, but it can be expected to be greater than that observed in the private sector [8]. Concentration of health expenditure in the public sector is probably at least partially related to emergency hospital admissions in 2008. These hospitalisations and the associated expenditure can be related to a complication of undiagnosed diabetes or another disease, leading, in both cases, to the diagnosis of type 2 diabetes during the hospitalisation.

A small acceleration in the growth of expenditure is observed in 2014 and 2015 for both cases and controls. Only analysis of expenditure after 2015 will be able to confirm this tendency and determine whether it is due to treatment intensification, improved medical follow-up, or the development of complications of diabetes or other outcomes.

Comparison of excess costs estimated by simple differences and DiD methods shows that the estimated excess cost varied only slightly according to the method used. First, the DiD method eliminates an initial difference in 2007 between the both groups in terms of expenditure. As a result of matching, this difference was minimal, except in the case of private-sector hospitalisations. Second, the DiD method allows the use of several individual characteristics as control variables. The minimal difference observed between the results estimated by the two methods shows the limited impact of inclusion of these control variables, and, therefore, the good quality of matching and its ability to reduce bias.

### Limitations

As we cannot benefit from any laboratory test results, it is impossible to comprehensively identify the entire population with type 2 diabetes. To avoid this measure bias, an identification algorithm based on drug treatments is used. The diabetic patients are, therefore, treated diabetics, which leads to the exclusion of all non-treated diabetic patients. Similarly, inpatients who have not yet received at least three antidiabetic treatments are not considered in the identification algorithm.

The algorithm is designed to accurately approximate the incident population of type 2 diabetics, but it requires a compromise between sensitivity and specificity. In particular, the database cannot distinguish between patients with type 2 diabetes and patients with the other types of diabetes (type 1 diabetes, LADA, steroid-induced, etc.). To exclude the greatest possible proportion of patients with type 1 diabetes, it was decided to exclude all patients under the age of 45 years in 2008. This choice leads to the exclusion of the youngest patients with type 2 diabetes. Estimates of expenditure, therefore, apply to a population aged 45 years and older, and not to younger individuals, who probably present different health expenditure profiles.

SHI databases also only include people reimbursed for health expenditure and individuals with no healthcare consumption are not present in this system. By and large, this causes to the exclusion of specific subpopulations, introducing a potential selection bias. For example, non-treated people, whether or not they are diabetic, may be non-treated for many different reasons (problems of access to care, refusal of care, and lack of private insurance), suggesting that they do not correspond to a standard profile and are disadvantaged population.

The matching and control variables are lagged variables and they should not have been influenced by the treatment. Health care preferences and habits could be a potential source of endogeneity, if they can impact both inclusion and treatment effect. We assume that health preferences are fixed over time, and so neutralized by DiD, which is consistent with the empiric literature [3]. The matching variable “risk factors” should capture lifestyle habits and risk factors effects (like overweight) involved in the onset of type 2 diabetes, which can impact the reimbursed expenditures. We also used a proxy from social background and place of residence, so the healthcare access constraints should be identical between both groups.

Due to the absence of public-sector MSO hospitalisation expenditure prior to 2008, these hospitalisations cannot be taken into account in the DiD method and the excess cost related to the onset of treated type 2 diabetes, therefore, cannot be measured by this methodological approach. Expenditure data are also not available for psychiatry, aftercare and rehabilitation, outpatient consultations and procedures, and hospital at home in the public sector prior to 2012. However, determination of the excess cost by simple differences for these types of expenditure from 2012 onwards shows that the excess cost is marginal compared to the excess cost related to MSO hospitalisations.

Matching allows measurement of the effect of treatment on treated subjects. The excess cost measured, therefore, corresponds to that induced by diabetes in a particular subsample of the general population: older, more frequently ill, and consuming reimbursed healthcare each year.

The similarity between groups is a necessary condition to attribute costs to the treated diabetes. That is why, we perform a matching method. Nevertheless, we can see that, before 2008, there is a small time difference in reimbursed expenditure between cases and controls, which is limiting the conclusion that can be drawn from the simple difference. These differences are more important for some items, but our study has the advantage to present results at a specific level, rather than an analysis by major items. The DiD should allow to correct the pre-existing difference between cases and control.

Parallel trend assumption is an identifying condition for DiD. It requires that, in the absence of treatment, the difference between the ‘treatment’ and ‘control’ group is constant over time. This requirement appears to be verified for primary care, but is more questionable for hospitalisations in private sector and cash benefits, so the results should be taken carefully. The smallest and least diabetes-related expenditure items diverge the most between case and control before treatment. For primary cares, the results are similar between simple difference and DiD.

Finally, standard errors of measured excess costs could be estimated by bootstrap methods. However, due to the large volume of data and the long processing times, this method cannot be used in this study. The risk of wrongly rejecting the null hypothesis (i.e., absence of excess cost induced by the incidence of treated type 2 diabetes) is minimal in reference to the large control group (more than 500,000 controls) and the effect size. There is no uncertainty about the reimbursed expenses to case, because we used all the incident population, and not a sample.

A test of robustness is performed by including a set of combinations of control variables in the DiD method. This test shows that the variables used have an extremely low impact, demonstrating the quality of matching and the comparability of the treated and control groups.

### Prospects

In the light of this study, it appears relevant to study the costs of diabetes in various ways. On one hand, costs could be studied by dividing the cohort into various subgroups (especially by age-group and by sex) to identify differences of trends according to the age of onset of diabetes, for example. The growth of costs could also be studied in perspective with the course of the disease and progress in treatment. Available healthcare consumption data can be used to estimate the patient’s health status and, therefore, study the links between health expenditure and health status. For example, we could distinguish patients in whom diabetes is diagnosed early and who initiated treatment with metformin monotherapy from patients in whom diabetes was diagnosed at the time of a complication. Detailed analysis of changes in treatment regimens and their impact in terms of expenditure could also be considered, especially to provide a better understanding of the factors responsible for the increased drug expenditure. One solution to avoid the problem of non-comparability of the excess cost estimated from 1 year to the next, due to death of members of the cohort, could be to estimate the excess cost in patients still alive in 2015, and to perform separate estimates for patients who died during the period, for example by year of death.

The continuing growth of excess cost for the cohort of incident patients in 2008 is likely to continue after 2015. This long-term study will be able to determine whether the increased expenditure in 2014–2015 is temporary or permanent and to observe the impact of the onset of complications related to type 2 diabetes on the estimated excess cost.

Finally, it could be interesting to reproduce this study on a new cohort of more recent diabetic patients (e.g.: 2013), especially to determine to what degree the arrival of new treatments, often more expensive, has been able to modify the expenditure attributed to diabetes.

## Conclusion

The mean reimbursed expenditure for a person with newly diagnosed type 2 diabetes is €4700 in 2008, for all types of expenditure. The mean diabetes-specific reimbursed expenditure (antidiabetic drugs, medical devices and services intrinsically related to diabetes and blood glucose, and glycosylated haemoglobin assays) is €220 per patient in 2008.

The use of difference-based methods of analysis can detect reimbursed expenditure which is not specific to diabetes, but related to the disease or its complications. A control group and DiD analysis are used to estimate the proportion of the total expenditure reimbursed to a person newly treated for diabetes.

The excess cost associated with the incidence of treated type 2 diabetes increases by an average of 4% per year. This excess cost is mainly due to primary care expenditure, essentially related to drugs, nursing care, and medical devices and services. The excess cost related to hospitalisation decreases during the year following the year of incidence of treated type 2 diabetes, and then increased again from the third year.

The excess cost, estimated by simple differences, is €1500 per patient and per year (for the first 7 years of the disease): from €1500 in the first year to €1800 in the seventh year. The DiD findings are similar to those obtained by simple differences, reflecting the robustness of the results obtained.

The study provides useful information for decision-makers concerning the dynamics of diabetes-related expenditure, which is very poorly documented in the scientific literature, particularly in France. The study of health expenditure reimbursed to the diabetic patients of the cohort will be continued after 2015 to study the future growth of excess costs related to diabetes and the development of complications.

## References

[1] Akobundu E, Ju J, Blatt L, Mullins CD (2006). Cost-of-illness studies. Pharmacoeconomics.

[2] Boyle JP, Thompson TJ, Gregg EW, Barker LE, Williamson DF (2010). Projection of the year 2050 burden of diabetes in the US adult population: dynamic modeling of incidence, mortality, and prediabetes prevalence. Popul. Health Metr..

[3] Chao LW, Szrek H, Pereira NS, Pauly MV (2009). Time preference and its relationship with age, health, and survival probability. Judgm. Decis. Mak..

[4] De Lagasnerie G, Aguadé A-S, Denis P, Fagot-Campagna A, Gastaldi-Ménager C (2017). The economic burden of diabetes to French national health insurance: a new cost-of-illness method based on a combined medicalized and incremental approach. Eur. J. Health Econ..

[5] Fagot-Campagna A, Romon I, Fosse S, Roudier C (2010). Prévalence et incidence du diabète, et mortalité liée au diabète en France—Synthèse épidémiologique.

[6] Fonds, C.M.U.: Rapport d’activité 2008. Fond de financement de la protection complémentaire de la couverture universelle du risque maladie, p. 8 (2008). http://www.cmu.fr/fichier-utilisateur/fichiers/Rapport_activite_2008.pdf. Accessed 19 July 2016

[7] Givord P (2014). Méthodes économétriques pour l’évaluation de politiques publiques. Économie & Prévision.

[8] Johnson JA, Pohar SL, Majumdar SR (2006). Health care use and costs in the decade after identification of type 1 and type 2 diabetes: a population-based study. Diabetes Care.

[9] Marcellusi A, Viti R, Mecozzi A, Mennini FS (2016). The direct and indirect cost of diabetes in Italy: a prevalence probabilistic approach. Eur. J. Health Econ..

[10] Mata-Cases M, Casajuana M, Franch-Nadal J, Casellas A, Castell C, Vinagre I (2016). Direct medical costs attributable to type 2 diabetes mellitus: a population-based study in Catalonia, Spain. Eur. J. Health Econ..

[11] NCD Risk Factor Collaboration (2016). Worldwide trends in diabetes since 1980: a pooled analysis of 751 population-based studies with 4·4 million participants. Lancet.

[12] Ortegon MM, Redekop WK, Niessen LW (2004). Cost-effectiveness of prevention and treatment of the diabetic foot: a Markov analysis. Diabetes Care.

[13] Pichetti S, Sermet C, van der Erf S (2013). The diffusion of new anti-diabetic drugs: an international comparison. Questions d’économie de la santé.

[14] Rey G, Rican S, Jougla E (2011). Mesure des inégalités de mortalité par cause de décès. Approche écologique à l’aide d’un indice de désavantage social. Bull. Epidemiol. Hebd. (Paris).

[15] Ricci P, Blotière PO, Weill A, Simon D, Tuppin P, Ricordeau P, Allemand H (2010). Diabète traité: quelles évolutions entre 2000 et 2009 en France. Bull. Epidemiol. Hebd. (Paris).

[16] Rubin DB (1974). Estimating causal effects of treatments in randomized and nonrandomized studies. J. Educ. Psychol..

[17] Seuring T, Archangelidi O, Suhrcke M (2015). The economic costs of type 2 diabetes: a global systematic review. Pharmacoeconomics.

[18] Sittig DT, Friedel H, Wasem J (2015). Prevalence and treatment costs of type 2 diabetes in Germany and the effects of social and demographical differences. Eur. J. Health Econ..

[19] Tuppin P, Rudant J, Constantinou P, Gastaldi-Ménager C, Rachas A, de Roquefeuil L (2017). Value of a national administrative database to guide public decisions: from the Système National d’Information Interrégimes de l’Assurance Maladie (SNDS) to the Système National des Données de Santé (SNDS) in France. Rev. Epidemiol. Sante Publique.

[20] Tao B, Pietropaolo M, Atkinson M, Schatz D, Taylor D (2010). Estimating the cost of type 1 diabetes in the US: a propensity score matching method. PLoS One.

